# Supplementary material for: Stabilizing a mammalian RNA thermometer confers neuroprotection in subarachnoid hemorrhage
Source: Nat Commun. 2025 Sep 18;16:8319. doi: 10.1038/s41467-025-63911-3 (PMC12446472; doi:10.1038/s41467-025-63911-3)
Supplement: Supplementary file 1 — Supplementary Information [file 41467_2025_63911_MOESM1_ESM.docx]

**Supplementary Materials for**

**Stabilizing a mammalian RNA thermometer confers neuroprotection in subarachnoid hemorrhage**

Min Zhang^1,2,3,#,^*, Bin Zhang^4,5,6,#^, Chengli Liu^7,#^, Marco Preußner^1^, Megha Ayachit^8^, Weiming Li^9^, Yafei Huang^10,11^, Deyi Liu^12^, Quanwei He^13^, Ann-Kathrin Emmerichs^1^, Stefan Meinke^1^, Shu Chen^14^, Lin Wang^15^, Liduan Zheng^16^, Qiubai Li^17,18^, Qin Huang^19^, Tom Haltenhof^1^, Ruoxi Gao^20^, Xianan Qin^21^, Aifang Cheng^22,23^, Tianzi Wei^24^, Li Yu^3^, Mario Schubert^8^, Xin Gao^4,5,6^, Mingchang Li^7,^*, Florian Heyd^1,^*

^1^Institut für Chemie und Biochemie, RNA Biochemie, Freie Universität Berlin, Berlin, Germany.

^2^Guangdong Key Laboratory for Biomedical Measurements and Ultrasound Imaging, National-Regional Key Technology Engineering Laboratory for Medical Ultrasound, School of Biomedical Engineering, Shenzhen University Medical School, Shenzhen University, Shenzhen, China.

^3^Department of Hematology and Oncology, Shenzhen University General Hospital, International Cancer Center, Hematology Institution, Shenzhen University Medical School, Shenzhen University, Shenzhen, China.

^4^Computer Science Program, Computer, Electrical and Mathematical Sciences and Engineering Division, King Abdullah University of Science and Technology (KAUST), Thuwal, Kingdom of Saudi Arabia.

^5^Center of Excellence for Smart Health (KCSH), King Abdullah University of Science and Technology (KAUST), Thuwal, Kingdom of Saudi Arabia.

^6^Center of Excellence on Generative AI, King Abdullah University of Science and Technology (KAUST), Thuwal, Kingdom of Saudi Arabia.

^7^Department of Neurosurgery, Renmin Hospital of Wuhan University, Wuhan, China.

^8^Institut für Chemie und Biochemie, Freie Universität Berlin, Berlin, Germany.

^9^Department of Hematology, Union Hospital, Tongji Medical College, Huazhong University of Science and Technology, Wuhan, China.

^10^Department of Pathogen Biology, School of Basic Medicine, Tongji Medical College, Huazhong University of Science and Technology, Wuhan, China.

^11^State Key Laboratory for Diagnosis and Treatment of Severe Zoonotic Infectious Diseases, Huazhong University of Science and Technology, Wuhan, China.

^12^Department of Pathophysiology, School of Basic Medicine, Chongqing Medical University, Chongqing, China.

^13^Department of Neurology, Union Hospital, Tongji Medical College, Huazhong University of Science and Technology, Wuhan, China.

^14^Department of Cardiovascular Surgery, Union Hospital, Tongji Medical College, Huazhong University of Science and Technology, Wuhan, China.

^15^Department of Pediatrics, Union Hospital, Tongji Medical College, Huazhong University of Science and Technology, Wuhan, China.

^16^Department of Pathology, Union Hospital, Tongji Medical College, Huazhong University of Science and Technology, Wuhan, China.

^17^Department of Rheumatology and Immunology, Union Hospital, Tongji Medical College, Huazhong University of Science and Technology, Wuhan, China.

^18^Hubei Engineering Research Center for Application of Extracelluar Vesicles, Hubei University of Science and Technology, Xianning, China.

^19^Department of Rehabilitation Medicine, Union Hospital, Tongji Medical College, Huazhong University of Science and Technology, Wuhan, China.

^20^Geriatric Medicine Center, Department of Geriatric Medicine, Zhejiang Provincial People’s Hospital (Affiliated People’s Hospital), Hangzhou Medical College, Hangzhou, China.

^21^School of Materials Science and Engineering, Zhejiang Sci-Tech University, Hangzhou, China.

^22^Department of Biomedical Sciences, Faculty of Health Sciences, University of Macau, Taipa, Macao SAR, China.

^23^Ministry of Education Frontiers Science Center for Precision Oncology, University of Macau, Taipa, Macao SAR, China.

^24^Department of Medical Neuroscience, School of Medicine, Southern University of Science and Technology, Shenzhen, China.

^#^These authors contributed equally

*Corresponding author. Min Zhang: minzhang900204@gmail.com; Mingchang Li: [mingcli@whu.edu.cn](mailto:mingcli@whu.edu.cn); Florian Heyd: florian.heyd@fu-berlin.de.


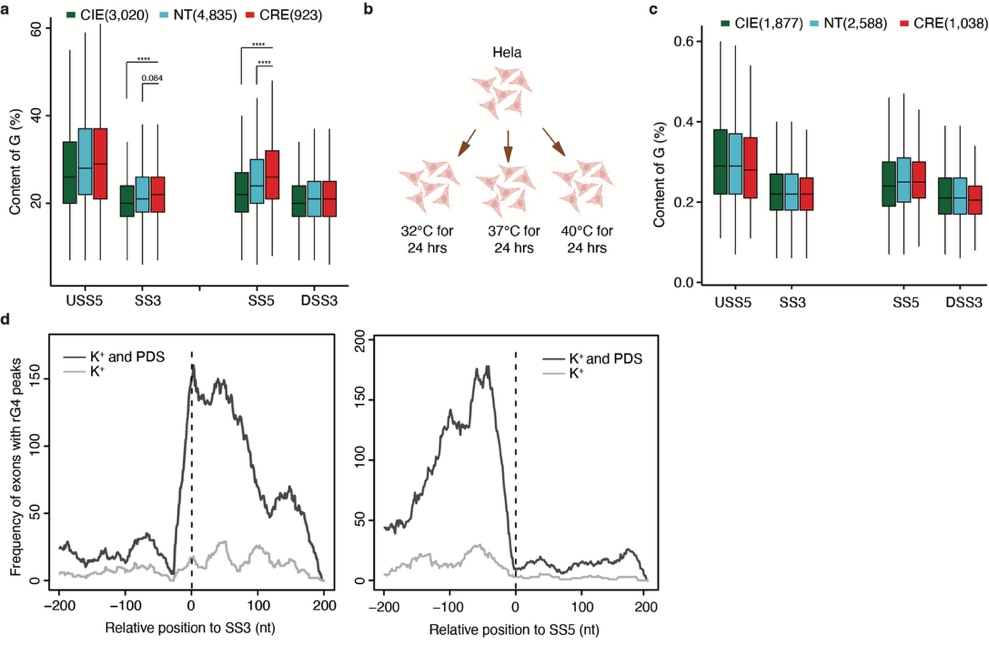
**Supplementary Fig. 1: G4 motifs are enriched around splice sites of cold-repressed exons (CRE).**

a Distribution of G-content in sequences within four regions (Fig. 1b) of CRE, CIE and NT in HEK293T cells (35°C vs. 39°C). The sample number of CIE (n=3020), NT(n=4835) and CRE (n=923) were indicated in the figure. The box displays the interquartile range (IQR) with the median line. Whiskers extend to the most extreme data points within 1.5×IQR of the box. Significance was estimated by Wilcox test and indicated where significant. ****, P < 0.0001.

b Schematic illustrating Hela cells cultured at 32°C, 37°C and 40°C.

c Distribution of G-content in sequence within four regions (Fig. 1b) of CRE, CIE and NT in Hela cells (37°C vs. 40°C). The sample number of CIE (n=1877), NT(n=2588) and CRE (n=1038) were indicated in the figure. The box displays the interquartile range (IQR) with the median line. Whiskers extend to the most extreme data points within 1.5×IQR of the box. Significance was estimated by Wilcox test and indicated where significant.

d The frequency of exons with rG4 peaks around splice sites of cassette exons from rG4-seq data of Hela cells treated with K^+^ and PDS or K^+^ alone (see bioinformatics Method).

**
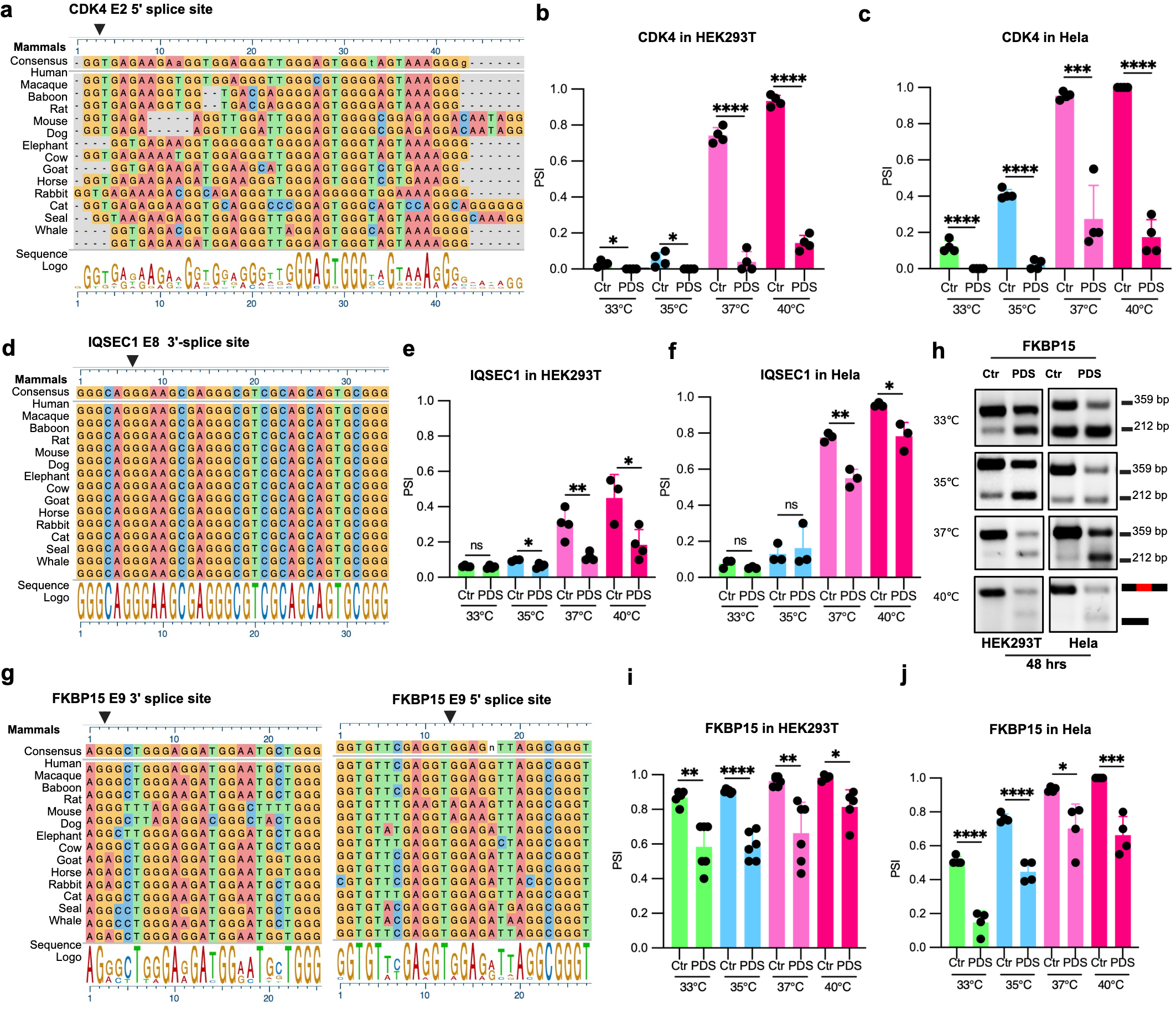
Supplementary Fig. 2: G4 stabilizers reduce the inclusion of cold-repressed exons.**

a, d and g The evolutionary conservation of G-rich motifs within the conserved regulatory elements (CREs) near splice sites across multiple mammalian species: (a) the 5′ splice site of CDK4 exon 2, (d) the 3′ splice site of IQSEC exon 8, and (g) both the 3′ splice site (left) and 5′ splice site (right) of FKBP15 exon 9, illustrated by a derived consensus sequence, the aligned sequences, and a sequence Logo representation (see bioinformatics method).

b, c, e, f, i and j Quantification of percent spliced-in (PSI) values for CREs in CDK4, IQSEC1, and FKBP15 in HEK293T (b, e, i) and HeLa cells (c, f, j) treated with DMSO or the G4 stabilizer PDS at various temperatures. panel b and c: n=4 independent biological replicates; panel e: n=3 (ctr at 35°C and 40°C), n=4 (others); panel f: n=3; panel i: n=5 (ctr at 33°C, PDS treatment at 40°C); n=4 (ctr at 40°C ), n=6 (others); panel j: n=4. In panel b, p=0.0181 (ctr vs. PDS) at 33°C, p=0.0404 at 35°C, p<0.0001 at 37°C, p<0.0001 at 40°C, p<0.0001; in panel c, p=0.0003 (ctr vs. PDS) at 33°C,p<0.0001 at 35°C，p=0.0003 at 35°C，p<0.0001 at 35°C; in panel e, p=0.0115 (ctr vs. PDS) at 35°C，p=0.0048 at 37°C，p=0.0225 at 40°C; in panel f, p=0.0022 at 37°C, p=0.0177 at 40°C; in panel i: p=0.0013 (ctr vs. PDS) at 33°C，p<0.0001 at 35°C，p=0.0022 at 37°C，p=0.0126 at 40°C; in panel j: p<0.0001 (ctr vs. PDS) at 33°C，p<0.0001 at 35°C，p=0.0191 at 37°C，p=0.0009 at 40°C.

h Representative RT-PCR gel images showing the inclusion levels of FKBP15 CRE exon 9 after PDS treatment in HEK293T and HeLa cells. Quantification of these results is shown in panels i (HEK293T) and j (HeLa). Individual data points and mean ± SD are shown. In this figure, statistical analysis was performed using two-tailed unpaired t test. ns denotes no significance, * denotes P ≤ 0.05, ** denotes P ≤ 0.01, *** denotes P ≤ 0.001 and **** denotes P ≤ 0.0001. Source data are provided as a Source Data file.

**
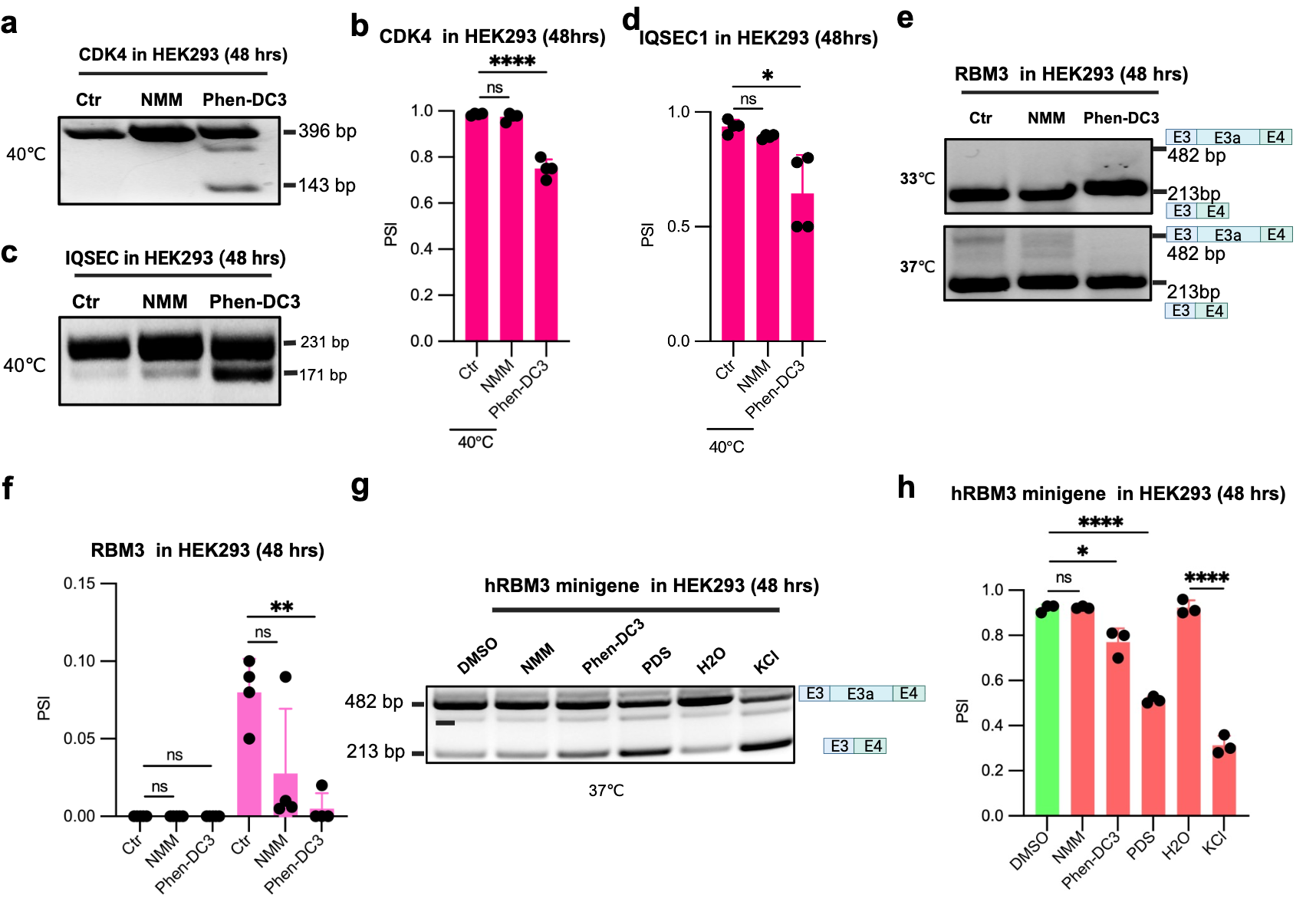
Supplementary Fig. 3: G4 ligands regulate alternative splicing of temperature sensitive rG4 containing exons in HEK293 cells.**

a, c, e and g Representative gel of CRE inclusion level of CDK4 (a), IQSEC1 (c), RBM3 (e) and RBM3 minigene (g) treated with DMSO or G4 ligands (10 µM for the NMM, Phen-DC3 and PDS, 50 mM for KCl) at indicated temperatures for 48 hrs in HEK293 cells. A representative gel image is shown. PCR products and sizes are indicated near the gel.

b, d, f and h Quantification results of a, c, e and g respectively. Individual data points and mean ± SD are shown. In panel b and d, n=4 independent biological replicates, p<0.0001 (Ctr vs. Phen-DC3) in CDK4 (b), p=0.0138 (Ctr vs. Phen-DC3) in IQSEC1(d) ; in panel f, n=5 at 33°C; n=4 at 37°C, p=0.0007 (Ctr vs. Phen-DC3 at 37°C); in panel h, n=3, p=0.0148 (DMSO vs. Phen-DC3), p<0.0001(DMSO vs. PDS), p<0.0001 (H_2_O vs. KCl). In this figure, statistical analysis was performed using two-tailed unpaired t test. ns denotes no significance, * denotes P ≤ 0.05, ** denotes P ≤ 0.01, **** P ≤ 0.001. Source data are provided as a Source Data file.


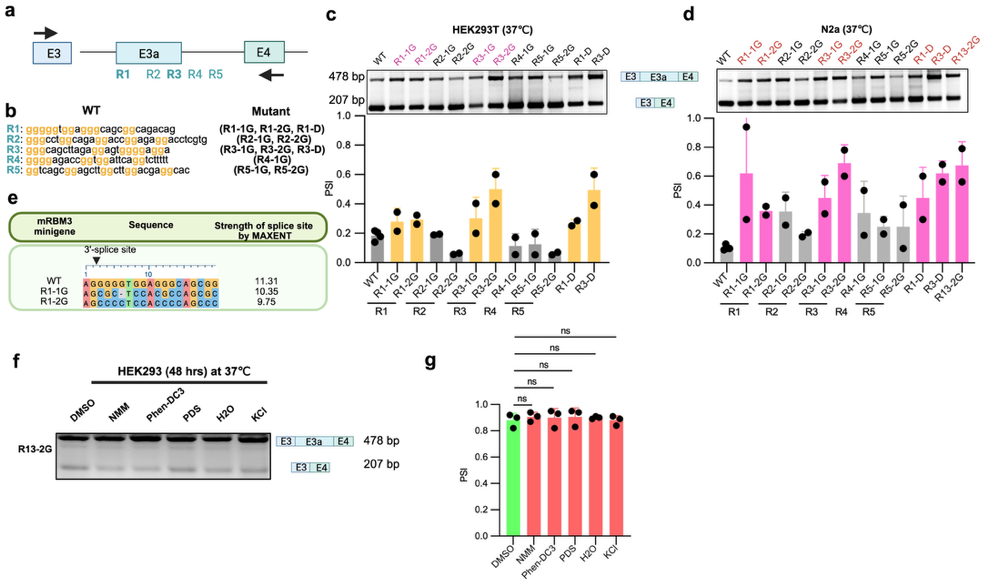
**Supplementary Fig. 4: Decreased inclusion of RBM3 exon 3a in the mutant of the conserved R1 and R3 elements.**

a Illustration of positions of G-rich elements adjacent to the splice sites of RBM3 exon 3a. R1, R2, R3, R4 and R5 represent the position of each G-rich element, respectively.

b WT sequences and their corresponding G-rich element mutants shown in (a). G-rich elements are highlighted in yellow. Constructs labeled R1-1G, R2-1G, R3-1G, R4-1G, and R5-1G contain a single G-to-C mutation in each G4 tract relative to the corresponding WT sequence. Constructs R1-2G, R2-2G, R3-2G, R4-2G, and R5-2G carry GG-to-CC mutations in each G4 tract.

c-d Inclusion levels of RBM3 exon 3a in WT and mutant mRBM3 minigenes at 37°C in HEK293T (c) and N2a (d) cells (n=2 independent biological replicates). A representative gel image is shown in the upper part. PCR products and sizes are indicated on the left. Quantification results are shown below the gel.

e Splice site strength predictions for the 3’-splice site of RBM3 exon 3a WT and mutants using MaxEntScan (see bioinformatics Method).

f Inclusion level of mRBM3 R13-2G minigene treated with DMSO or G4 ligands (10 µM for the PDS, Phen-DC3 and NMM, 50 mM for KCl) at 37°C for 48 hrs in HEK293 cells (n=3 independent biological replicates). A representative gel image is shown. PCR products and sizes are indicated on the right.

g Quantification results of f. Individual data points and mean ± SD are shown (n=3 independent biological replicates). In this figure, statistical analysis was performed using two-tailed unpaired t test. ns denotes no significance. Source data are provided as a Source Data file.


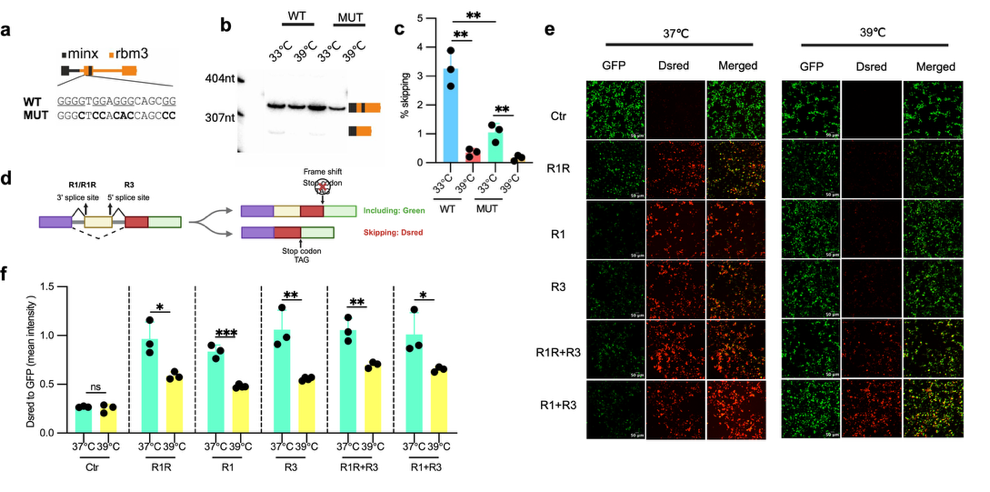
**Supplementary Fig. 5: G-rich elements regulate temperature-sensitive alternative splicing.**

a Schematic of MINX minigenes containing part of the RBM3 pre-mRNA. A segment of the RBM3 gene starting with exon 3a, including either the WT or mutated G-rich R1 element, was cloned in the MINX minigene. The construct contains only the splice sites and G-rich sequences within exon 3a but no other exonic splicing splicing regulatory sequences. MINX sequence is highlighted in black, RBM3 sequence in orange.

b Representative splicing results of the MINX minigenes from (a) by radioactive RT-PCR (n=3 independent biological replicates). The minigenes were transfected into HEK293T cells overnight, followed by different temperature treatments for another 24hrs.

c Quantification of (b), individual data points and mean ± SD are shown (n=3 independent biological replicates). p=0.0014 (33°C vs. 39°C for WT)，p=0.0093 (33°C vs. 39°C for MUT).

d Schematic of the fluorescent minigene reporter containing R1 and/or R3 G-rich elements. The R1 and/or R3 elements were cloned near the splice sites of the cassette exon of the reporter gene^1^. The inserted cassette exon introduces a frameshift in DsRed and lacks a stop codon upstream of GFP, resulting in GFP expression but no DsRed expression. However, if the cassette is skipped, DsRed is expressed, and the stop codon prevents GFP expression. R1R denotes the replacement of the sequence near the splice sites of the cassette exon in the minigene with R1, while the others represent the insertion of R1 and/or R3 near the splice sites.

(e) Reprehensive confocal images of HEK293 cells transfected with different fluorescent minigene reporters from (d). The minigenes were transfected into HEK293 cells overnight, followed by different temperature treatment for another 24hrs.

(f) Quantification of images in (e). The mean fluorescent intensity in each image was automatically quantified by Fiji. Individual datapoints and mean ± SD are shown (n=3 independent biological replicates). p=0.5786 (37°C vs. 39°C) for WT, p=0.0208 for R1R, p=0.0001 for R1, p=0.0036 for R3, p=0.0081 for R1R+R3, p=0.0495 for R1+R3. Statistical analysis was performed using two-tailed unpaired t test. ns denotes no significance, * denotes P ≤ 0.05, ** denotes P ≤ 0.01, ***P ≤ 0.001. Source data are provided as a Source Data file.

**
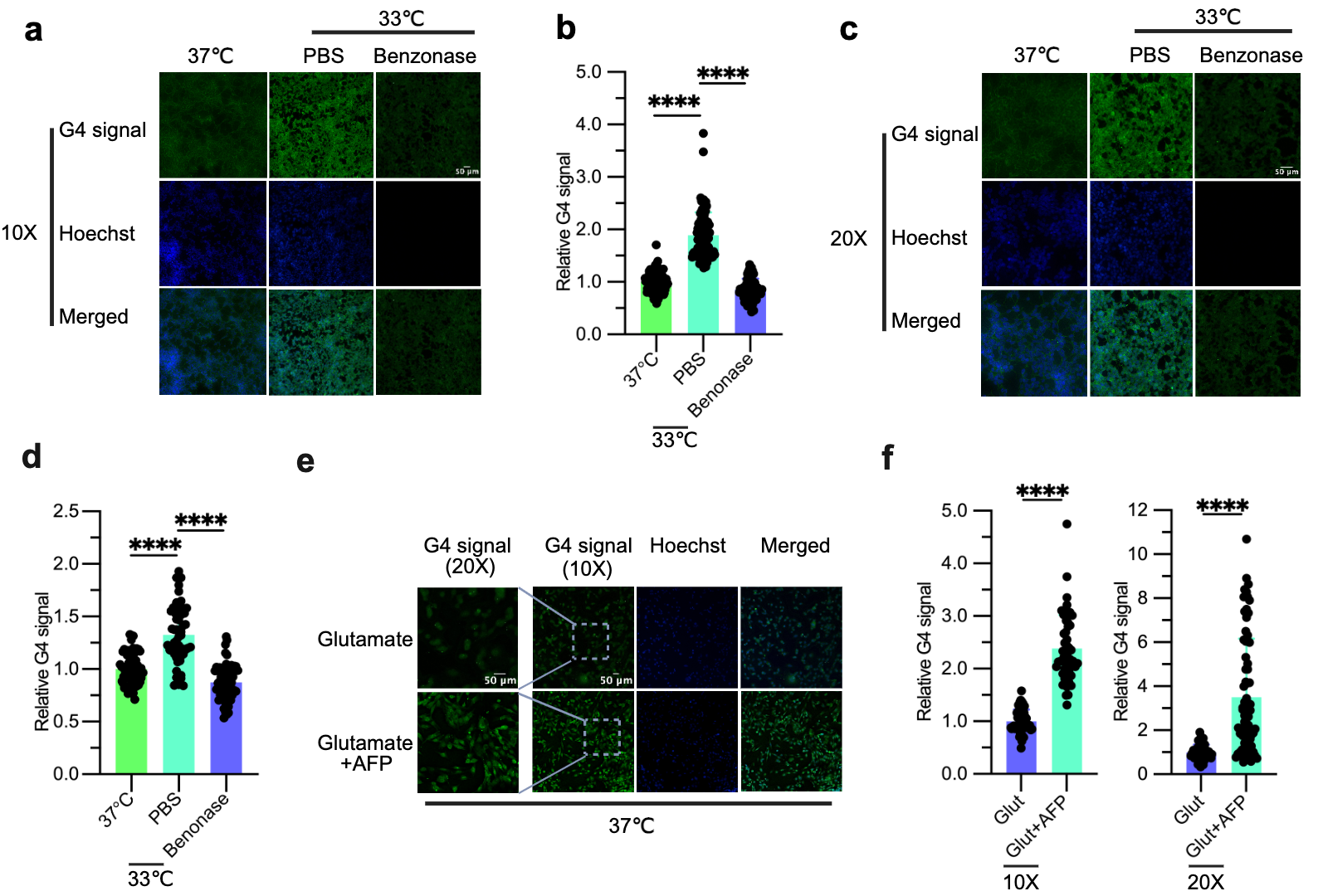
Supplementary** **Fig. 6:** **G4-specific immunostaining shows temperature and potassium dependent G-quadruplex formation in live cells.**

a, c and e Representative confocal images showing G4-specific staining under different temperatures and treatment conditions in HEK293 cells (a, c) and HT22 cells (e). Panel (a) presents 10× magnification images, while panel (b) shows corresponding 20× magnification images, as indicated in the figure. Cells were treated at different temperatures for 48hrs, followed by PBS or benzonase treatment and immunostaining with G4 specific antibody and Hoechst, then confocal imaging. Glutamate treatment can induce membrane depolarization in HT22 cells, activating the voltage-gated potassium channel and can mimic the neuronal injury^2^. Voltage-gated potassium channel blocker (AFP, Amifampridine) is used to increase intracellular potassium in the glutamate-depolarized/-excited HT22 cells.

b, d and f Quantifications of (a), (c) and (e) respectively with Fiji. Quantifications were based on three independent biological replicate images, with signal intensity measured in 18-32 cells (in b: n=32 cells at 37°C, n=30 cells at 33°C (PBS), n=21 cells at 33°C (Benzonase) per image; in d: n=21 cells at 37°C, n=18 cells at 33°C (PBS), n=22 cells (Benzonase); in f: for 10X magnification (n=17 cells for Glut and Glut+AFP), for 20X magnification (n=20 cells for Glut, n=25 cells for Glut+AFP). p<0.0001 in all comparisons in this figures. Each dot represents the average intensity of G4 in a single cell. The scatter dot plots are shown as the mean ± SD. Each individual data point is shown. Statistical analysis was performed using two-tailed unpaired t test. **** denotes P ≤ 0.0001. Source data are provided as a Source Data file.

**
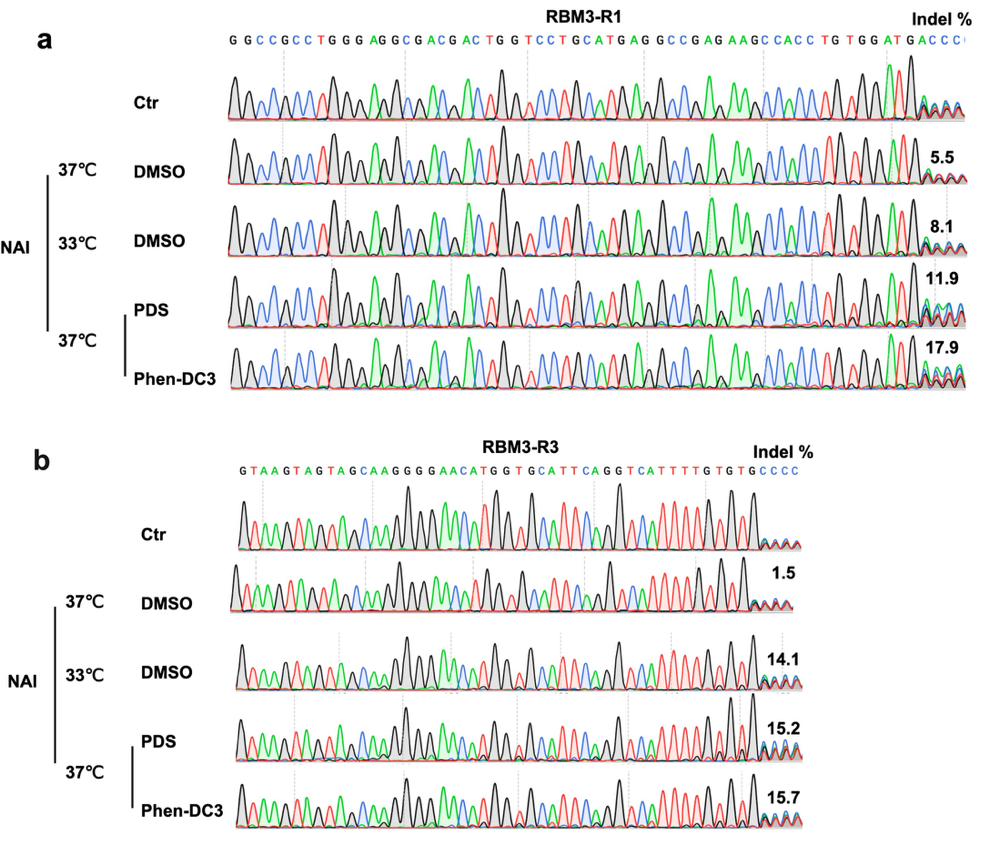
Supplementary Fig. 7: SHAPE-MaP probing shows temperature dependent G-quadruplex formation of RBM3 exon3a in living cells.**

a and b Representative sanger sequencing results of gel-purified SHAPE-modified products near the 5’ (RBM3-R1) and 3’ (RBM3-R3) splice sites of RBM3 cold-repressed exon 3a containing putative rG4. The indel percentage labeled in the figure is quantified by TIDE software^3^. Source data are provided as a Source Data file.

**
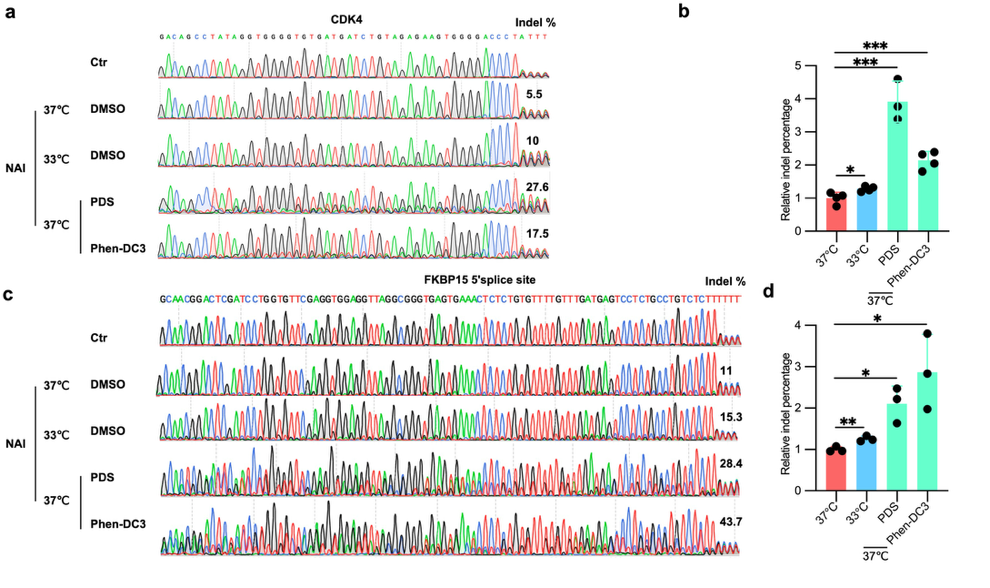
Supplementary Fig. 8: SHAPE-MaP probing shows temperature-dependent G-quadruplex formation of CDK4 and FKBP15 in living cells.**

a and c Representative sanger sequencing results of gel-purified SHAPE-modified products near the splice sites of cold-repressed exons containing putative rG4 in CDK4 and FKBP15.

b and d Quantified indel percentage of sanger sequencing data from (a and c). The indel percentages were quantified using TIDE software^3^ within the 50 bp upstream and downstream regions of the putative rG4^3^. In b, n=3 independent biological replicates for PDS, n=4 for others, p=0.0280 (37°C vs. 33°C), p= 0.0003 (37°C vs. PDS)，p=0.0004 (37°C vs. Phen-DC3); in d, n=3, p=0.0099 (37°C vs. 33°C), p=0.0271(37°C vs. PDS)，p=0.0241 (37°C vs. Phen-DC3). The scatter dot plots are shown as the mean ± SD. Statistical analysis was performed using two-tailed unpaired t test. * denotes P ≤ 0.05, ** denotes P ≤ 0.01 and ***P ≤ 0.001. Source data are provided as a Source Data file.


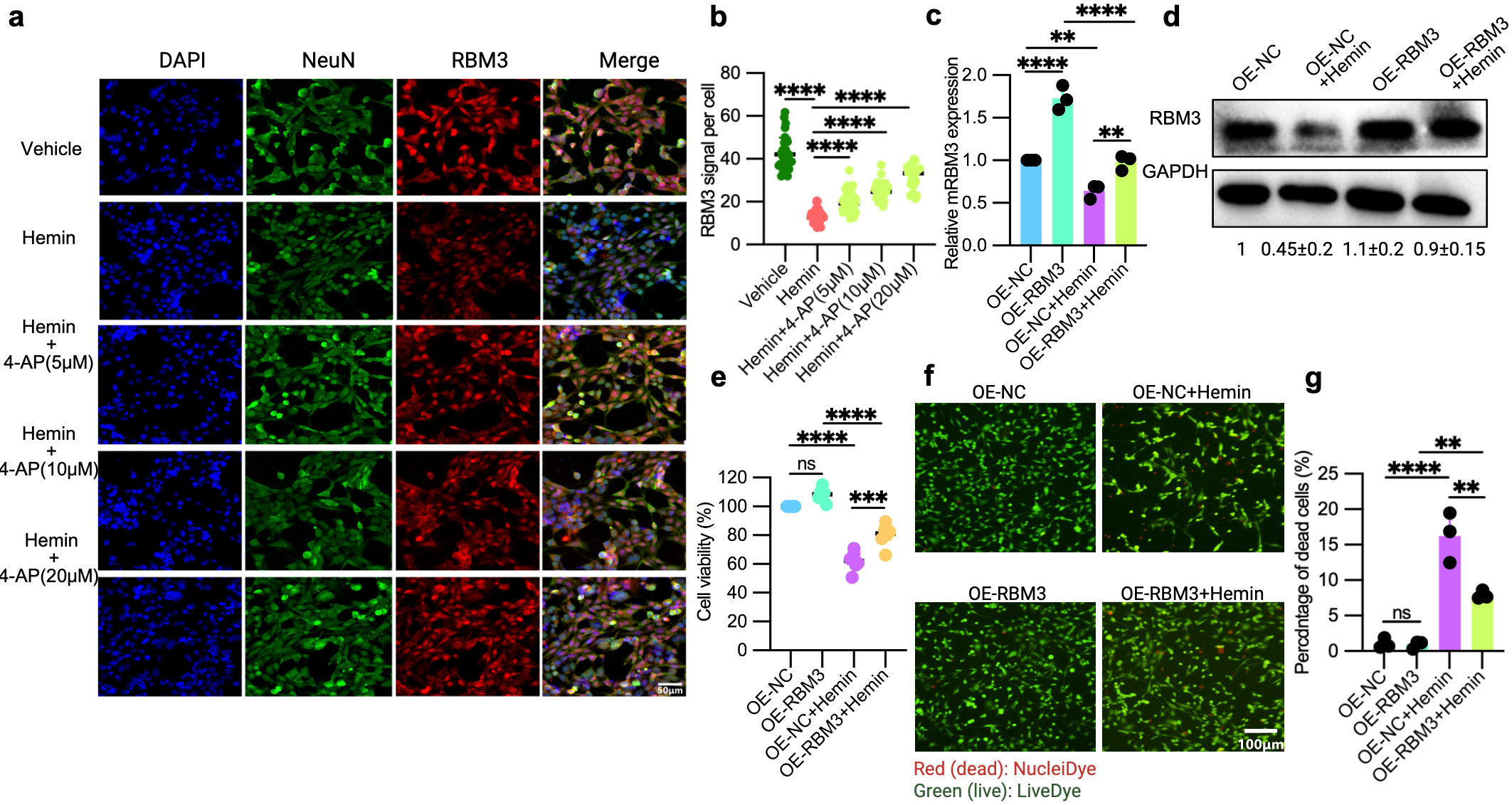
 **Supplementary Fig. 9: RBM3-mediated neuronal protection by 4-AP in the hemin-induced cell model.**

a Representative immunostaining of nuclear marker DAPI, neuronal marker NeuN, RBM3 and merged images from treatments as in Fig. 6a.

b Relative RBM3 expression was quantified in images as shown in (a) (n=3 independent biological replicate images). p<0.0001 in all comparisons.

c and d Relative RBM3 expression after lentiviral overexpression in hemin- and control-treated HT22 cells. RBM3 mRNA expression was assessed by qPCR in (c) (n=3 independent biological replicates), while RBM3 protein expression was measured by Western blot in (d). Below the gel, quantification relative to GAPDH is shown (see Method, n=3 independent biological replicates). In c: p<0.0001 (OE-NC vs. OE-RBM3, OE-RBM3 vs. OE-RBM3+Hemin), p=0.0048 (OE-NC vs. OE-NC+Hemin), p=0.0067 (OE-NC+Hemin vs. OE-RBM3+Hemin); In d: p=0.0019 (OE-NC vs. OE-NC+Hemin), p= 0.0104 (OE-NC+Hemin vs. OE-RBM3+Hemin).

e Cell viability in treatments as in (c), quantified by CCK-8 assay in HT22 cells (n=6 independent biological replicates). p<0.0001 (OE-NC vs. OE-NC+Hemin, OE-RBM3 vs. OE-RBM3+Hemin)， p=0.0001 (OE-NC+Hemin vs. OE-RBM3+Hemin)

f-g The percentage of dead cells in treatments as in (c). Stained images for dead/live cells are presented in (f), with quantification of dead cells in (g) (see Method, n=3 independent biological replicate images). p<0.0001 (OE-NC vs. OE-NC+Hemi), p=0.0050 (OE-RBM3 vs. OE-RBM3+Hemin)， p=0.0017 (OE-NC+Hemin vs. OE-RBM3+Hemin). In this figure, the scatter dot plots are shown as the mean ± SD. Statistical analysis was conducted using ordinary one-way ANOVA followed by Šídák’s multiple comparisons test (b: Dunnett’s multiple comparisons test) with a single pooled variance. ns denotes no significance, * denotes P ≤ 0.05, ** denotes P ≤ 0.01, ***P ≤ 0.001 and **** denotes P ≤ 0.0001. Source data are provided as a Source Data.

**
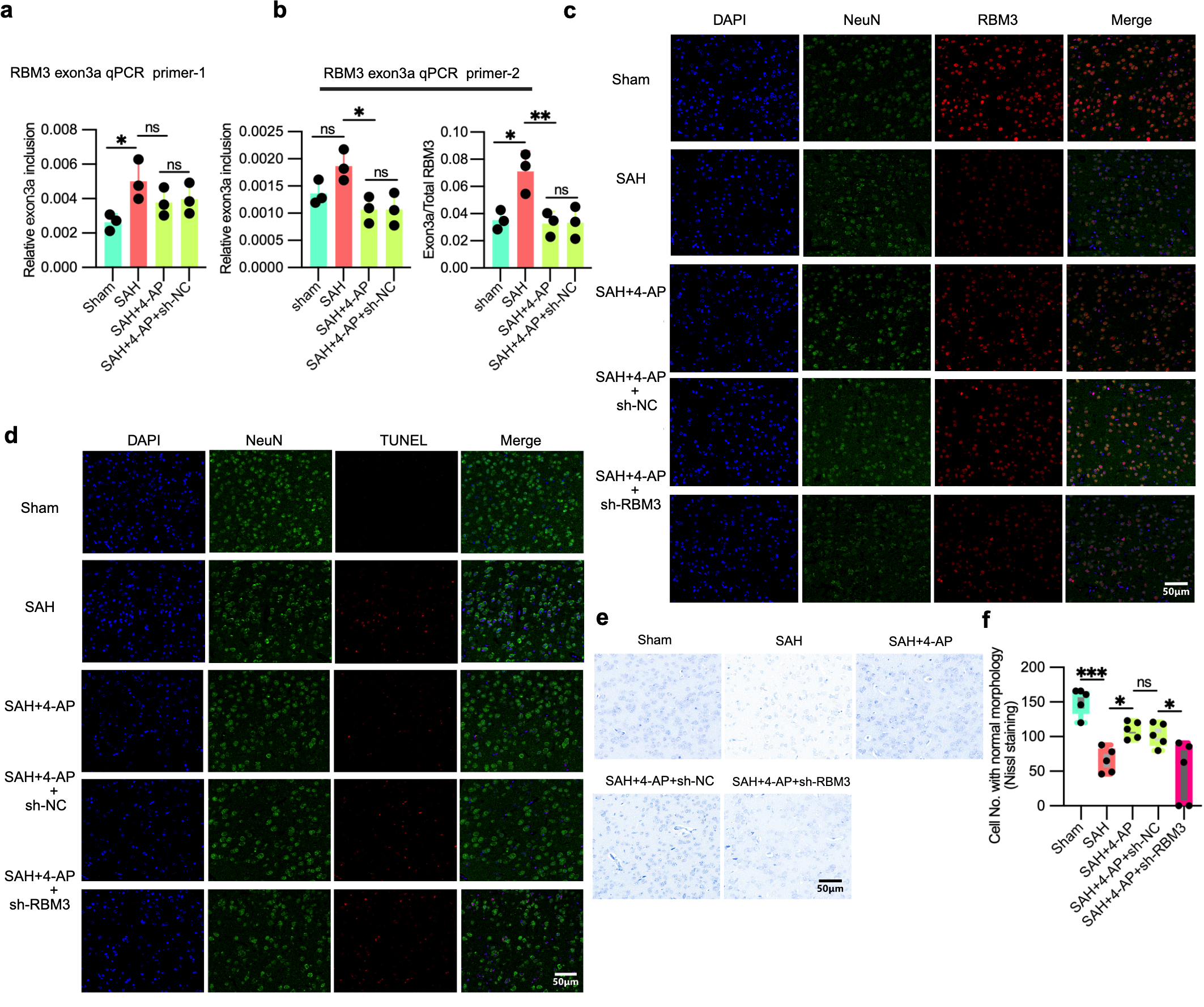
 Supplementary Fig. 10: RBM3-mediated neuronal protection by 4-AP in the SAH mouse model.**

a and b RBM3 exon 3a inclusion and the ratio of RBM3 exon 3a to total RBM3 following 4AP administration in the cortical region (Fig. 7b) of the SAH mouse model. RBM3 exon 3a qPCR primer-1 (a) and RBM3 exon 3a qPCR primer-2 (b) were used to quantify RBM3 exon 3a inclusion, respectively. In panel (a), mouse GAPDH was used as normalized control. In panel (b), the left graph shows RBM3 exon 3a expression normalized to mGAPDH, while the right graph displays the ratio of RBM3 exon 3a to total RBM3 transcript levels (see Method, n=3 mice). In panel a, p= 0.0312(Sham vs. SAH)， p=0.3229 (SAH vs. SAH+4-AP)， p=0.9910 (SAH+4-AP vs. SAH+4-AP+sh-NC); in panel b (left), p=0.1408 ( sham vs. SAH)， p=0.0185 (SAH vs. SAH+4-AP)，p>0.9999 (SAH+4-AP vs. SAH+4-AP+sh-NC); in panel b (right), p=0.0128 (sham vs. SAH), p =0.0086 (SAH vs. SAH+4-AP), p=0.9997 (SAH+4-AP vs. SAH+4-AP+sh-NC).

c Representative immunostaining images in the cortical region (Fig. 7b) of the SAH injected with lenti-shRBM3 and lenti-NC and sham mouse model in vivo after 4-AP and control administration. Quantification data is in Fig. 7f and h (also see Method, n=5 mice).

d Representative immunostaining images of DAPI, NeuN and TUNEL of the cortical region (Fig. 7b) in the mice treated as Fig. 7a. Quantification data is in Fig. 7g (n=5 mice).

e Representative Nissl-stained images of the cortical region (Fig. 7b) of the mice treated as described in Fig. 7a (n=5).

f Quantified number of Nissl substance-positive cells with normal morphology in (e) (n=5 mice). p=0.0001(Sham vs. SAH)，p=0.0456 (SAH vs. SAH+4-AP)，p=0.0113 (SAH+4-AP+sh-NC vs. SAH+4-AP+sh-RBM3), p=0.9948 (SAH+4-AP vs. SAH+4-AP+sh-NC). Supplementary Fig. 10f is presented as box and whisker plots with min to max showing all points of biological replicates. Others are the scatter dot plots shown as the mean ± SD. Statistical analysis was conducted using ordinary one-way ANOVA followed by Šídák’s multiple comparisons test with a single pooled variance. ns denotes no significance, * denotes p≤ 0.05, ** denotes p≤ 0.01, *** denotes p≤ 0.01. Source data are provided as a Source Data file.


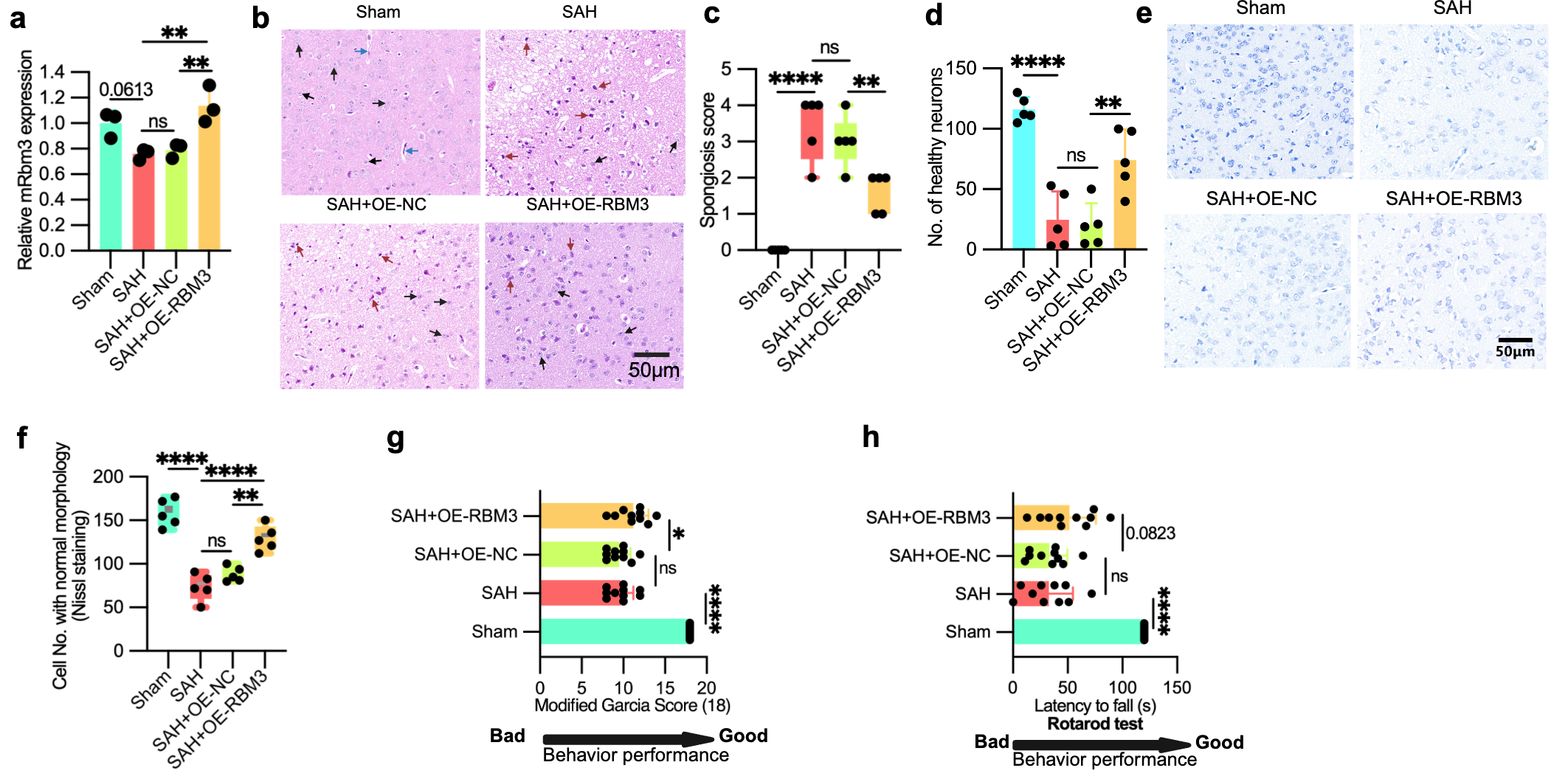
**Supplementary Fig. 11: Overexpression of RBM3 confers neuronal protection in the SAH mouse model.**

a RBM3 mRNA expression of the SAH injected with lenti-OE-RBM3 and lenti-NC and sham mouse model in vivo (also see Fig. 7a and Method, n=3 mice). p=0.0613 (Sham vs. SAH), p= 0.9917 (SAH vs. SAH+OE-NC)，p=0.0053 (SAH vs. SAH+OE-RBM3)，p=0.0090 (SAH+OE-NC vs. SAH+OE-RBM3).

b Representative HE stainings in the cortical region (Fig. 7b) of the mice treated as described in (a) and Fig. 7a. Representative healthy neurons are denoted by black arrows, damaged neurons by red arrows and endothelial cells in the capillary by blue arrows (n=5 mice).

c Quantified Spongiosis score of HE staining in (b) (n=5 mice). Spongiosis was scored on a scale of 0–4 based on the degree of intercellular edema and vesicle/bullae formation: 0=absent, 1= minimal, 2=mild, 3=moderate, 4=severe (more details, also see Method). p<0.0001 (Sham vs. SAH)， p=0.7022 (SAH vs. SAH+OE-NC) ，p=0.0089 (SAH+OE-NC vs. SAH+OE-RBM3).

d Healthy neuronal count of the HE staining shown in (b) (n=5 mice). Healthy neurons are identified by round or oval cell bodies with lightly eosinophilic cytoplasm, centrally located nuclei, and prominent nucleoli. Unhealthy neurons are identified by eosinophilic necrosis, exhibiting cell body shrinkage, intensely stained eosinophilic cytoplasm, and darkly stained pyknotic nuclei. p<0.0001 (Sham vs. SAH), p=0.9814 (SAH vs. SAH+OE-NC), p=0.0019 (SAH+OE-NC vs. SAH+OE-RBM3).

e Representative images of Nissl staining in the cortical region (Fig. 7b) of the mice treated as (a) (also see Fig. 7a and Method, n=5 mice).

f Quantified number of Nissl substance-positive cells with normal morphology in (e) (n=5 mice). p<0.0001 (Sham vs. SAH)， p=0.3843 (SAH vs. SAH+OE-NC)， p<0.0001 (SAH vs. SAH+OE-RBM3)， p=0.0011 (SAH+OE-NC vs. SAH+OE-RBM3).

g Modified Garcia score assessing neurological function in mice treated as (a). This composite score evaluates spontaneous activity, limb symmetry, forepaw outstretching, climbing, body proprioception, and response to vibrissae stimulation (see Method^55^, n=10 mice). p<0.0001 (Sham vs. SAH)， p=0.9831 (SAH vs. SAH+OE-NC)， p=0.0241 (SAH+OE-NC vs. SAH+OE-RBM3).

h Latency to fall of the mice treated as (a) in the Rotarod Test (see Method, n=10 mice). p<0.0001 (Sham vs. SAH)， p>0.9999 (SAH vs. SAH+OE-NC), p=0.0823 (SAH+OE-NC vs. SAH+OE-RBM3). Supplementary Fig. 11c and 11f are shown as box and whisker plots with min to max showing all points of biological replicates. Others are the scatter dot plots shown as the mean ± SD. Statistical analysis was conducted using ordinary one-way ANOVA followed by Šídák’s multiple comparisons test with a single pooled variance. ns denotes no significance, * denotes P ≤ 0.05, ** denotes P ≤ 0.01, and **** denotes P ≤ 0.0001. Source data are provided as a Source Data.

**Supplementary Table 1: Modified Garcia scoring system for neurological assessment in mice.**
This table outlines the components and scoring criteria used to assess neurological function following experimental intervention. Each parameter—spontaneous activity, limb movement symmetry, climbing ability, forelimb outstretching, touch response, and proprioception response—is scored on a scale of 0–3, with higher scores indicating better neurological function. Detailed descriptions for each score category are provided to ensure reproducibility and consistent evaluation across experiments.

**Reference**

1 Huang, H., Zhang, J., Harvey, S. E., Hu, X. & Cheng, C. RNA G-quadruplex secondary structure promotes alternative splicing via the RNA-binding protein hnRNPF. Genes Dev **31**, 2296-2309 (2017). <https://doi.org:10.1101/gad.305862.117>

2 Lai, K. et al. Glutamate acts on acid-sensing ion channels to worsen ischaemic brain injury. Nature **631**, 826-834 (2024). <https://doi.org:10.1038/s41586-024-07684-7>

3 Brinkman, E. K., Chen, T., Amendola, M. & van Steensel, B. Easy quantitative assessment of genome editing by sequence trace decomposition. Nucleic Acids Res **42**, e168 (2014). <https://doi.org:10.1093/nar/gku936>
