# Supplementary material for: Stabilizing a mammalian RNA thermometer confers neuroprotection in subarachnoid hemorrhage
Source: Nat Commun. 2025 Sep 18;16:8319. doi: 10.1038/s41467-025-63911-3 (PMC12446472; doi:10.1038/s41467-025-63911-3)
Supplement: Supplementary file 2 — Description of Additional Supplementary Files [file 41467_2025_63911_MOESM2_ESM.pdf]

Description of Additional Supplementary Files

Supplementary data 1 (Consensus Cold-Repressed Exons (CREs) in HEK293T and HeLa Cells Under Different temperatures): Summary of common cold-responsive exons (CREs) identified in both HEK293T and HeLa cells. In detail, CRE were defined as exons with ΔPSI $\leq$ -0.1 (for HEK293T: 35°C-39℃; for Hela: 32°C-37℃). Significance was assessed by a two-sided likelihood-ratio test with Benjamini-Hochbergadjusted p-value < 0.05 (for HEK293T: 35°C vs 39℃; for Hela: 32°C vs 37℃). The CREs listed in this table represent the intersection of CREs identified in both HEK293T and HeLa cells.

Supplementary data 2 (Key resources used in this study): This table lists all primary reagents, resources, and materials employed in the experiments, including antibodies, lentiviral particles, siRNAs, chemicals, buffers, and enzymes. Each entry specifies the reagent/resource name, the source or supplier, and the unique identifier or catalog number, allowing unambiguous reference and reproducibility of the study.
