# Supplementary material for: Stabilizing a mammalian RNA thermometer confers neuroprotection in subarachnoid hemorrhage
Source: Nat Commun. 2025 Sep 18;16:8319. doi: 10.1038/s41467-025-63911-3 (PMC12446472; doi:10.1038/s41467-025-63911-3)
Supplement: Supplementary file 5 — Reporting Summary [file 41467_2025_63911_MOESM5_ESM.pdf]

nature portfolio

Corresponding author(s): Min ZHANG

Last updated by author(s): Aug 6, 2025

Reporting Summary

Nature Portfolio wishes to improve the reproducibility of the work that we publish. This form provides structure for consistency and transparency in reporting. For further information on Nature Portfolio policies, see our Editorial Policies and the Editorial Policy Checklist.

Statistics

For all statistical analyses, confirm that the following items are present in the figure legend, table legend, main text, or Methods section.

n/a	Confirmed	
☐	☒	The exact sample size (n) for each experimental group/condition, given as a discrete number and unit of measurement
☐	☒	A statement on whether measurements were taken from distinct samples or whether the same sample was measured repeatedly
☐	☒	The statistical test(s) used AND whether they are one- or two-sided<br>*Only common tests should be described solely by name; describe more complex techniques in the Methods section.*
☒	☐	A description of all covariates tested
☐	☒	A description of any assumptions or corrections, such as tests of normality and adjustment for multiple comparisons
☐	☒	A full description of the statistical parameters including central tendency (e.g. means) or other basic estimates (e.g. regression coefficient) AND variation (e.g. standard deviation) or associated estimates of uncertainty (e.g. confidence intervals)
☒	☐	For null hypothesis testing, the test statistic (e.g. F, t, r) with confidence intervals, effect sizes, degrees of freedom and P value noted<br>*Give P values as exact values whenever suitable.*
☒	☐	For Bayesian analysis, information on the choice of priors and Markov chain Monte Carlo settings
☒	☐	For hierarchical and complex designs, identification of the appropriate level for tests and full reporting of outcomes
☒	☐	Estimates of effect sizes (e.g. Cohen's d, Pearson's r), indicating how they were calculated

Our web collection on statistics for biologists contains articles on many of the points above.

Software and code

Policy information about availability of computer code

Data collection	RNA-Seq for HEK293T: our previous published RNA-seq data in HEK293T cells at different temperatures (GEO #GSE143872.);<br>RNA-Seq for Hela: new generated (GEO #GSE262498)
Data analysis	The original codes are available in Github (https://github.com/christear/G4splicing) for rG4 analysis.<br>These following softwares are used for the data analysis:<br>rMATs (V3.1.0)<br>Python (jupyter Notebooks with Pythons version 3)<br>R (V4.2.1)<br>BEDTOOLS (V2.3)<br>G4Hunter (https://academic.oup.com/nar/article/44/4/1746/1854457)<br>DNASTAR (V17.3.0.57)<br>TIDE (https://apps.datacurators.nl/tide/)<br>GraphPad Prism (V10.4.1 (532))<br>Fiji (ImageJ2 V2.16.0/1.54g)

For manuscripts utilizing custom algorithms or software that are central to the research but not yet described in published literature, software must be made available to editors and reviewers. We strongly encourage code deposition in a community repository (e.g. GitHub). See the Nature Portfolio guidelines for submitting code & software for further information.

Data

Policy information about availability of data

All manuscripts must include a data availability statement. This statement should provide the following information, where applicable:

- Accession codes, unique identifiers, or web links for publicly available datasets
- A description of any restrictions on data availability
- For clinical datasets or third party data, please ensure that the statement adheres to our policy

RNA-Seq for Hela is made publically available under GSE262498. All raw data used for quantification and analysis are provided in the source data file or the supplementary materials.

Research involving human participants, their data, or biological material

Policy information about studies with human participants or human data. See also policy information about sex, gender (identity/presentation), and sexual orientation and race, ethnicity and racism.

Reporting on sex and gender	*Use the terms sex (biological attribute) and gender (shaped by social and cultural circumstances) carefully in order to avoid confusing both terms. Indicate if findings apply to only one sex or gender; describe whether sex and gender were considered in study design; whether sex and/or gender was determined based on self-reporting or assigned and methods used. Provide in the source data disaggregated sex and gender data, where this information has been collected, and if consent has been obtained for sharing of individual-level data; provide overall numbers in this Reporting Summary. Please state if this information has not been collected. Report sex- and gender-based analyses where performed, justify reasons for lack of sex- and gender-based analysis.*
Reporting on race, ethnicity, or other socially relevant groupings	*Please specify the socially constructed or socially relevant categorization variable(s) used in your manuscript and explain why they were used. Please note that such variables should not be used as proxies for other socially constructed/relevant variables (for example, race or ethnicity should not be used as a proxy for socioeconomic status). Provide clear definitions of the relevant terms used, how they were provided (by the participants/respondents, the researchers, or third parties), and the method(s) used to classify people into the different categories (e.g. self-report, census or administrative data, social media data, etc.) Please provide details about how you controlled for confounding variables in your analyses.*
Population characteristics	*Describe the covariate-relevant population characteristics of the human research participants (e.g. age, genotypic information, past and current diagnosis and treatment categories). If you filled out the behavioural & social sciences study design questions and have nothing to add here, write "See above."*
Recruitment	*Describe how participants were recruited. Outline any potential self-selection bias or other biases that may be present and how these are likely to impact results.*
Ethics oversight	*Identify the organization(s) that approved the study protocol.*

Note that full information on the approval of the study protocol must also be provided in the manuscript.

Field-specific reporting

Please select the one below that is the best fit for your research. If you are not sure, read the appropriate sections before making your selection.

☒ Life sciences ☐ Behavioural & social sciences ☐ Ecological, evolutionary & environmental sciences

For a reference copy of the document with all sections, see nature.com/documents/nr-reporting-summary-flat.pdf

Life sciences study design

All studies must disclose on these points even when the disclosure is negative.

Sample size	RNA-seq experiments were conducted using four independent biological replicates per treatment condition in Hela cells. For all experiments involving statistical analysis, a minimum of three independent biological replicates (for in vivo mouse experiments, 5 mice per treatment group) was used. While no formal sample size calculation was performed, these replicate numbers are consistent with standards in the field and have been demonstrated to provide sufficient power to detect biologically meaningful differences.
Data exclusions	No data is excluded.
Replication	All experiments for statistical analyses were conducted with at least three independent biological replicates. All attempts at replications were successful.
Randomization	For computational analyses, sample grouping was based on predefined experimental conditions, and random allocation was not applicable. Each treatment group included three or four biological replicates. For all rG4-related analyses, the upstream and downstream exons served as internal controls.<br>For biological experiments, groups were assigned based on treatment conditions. In RT-PCR experiments, a control without reverse

transcriptase was use as a negative control. A non-targeting or scrambled siRNA was used as a normalization control for all knockdown experiments, and empty vectors were included as within-batch controls for all overexpression studies.

For in vivo mouse experiments, animals were randomly assigned to treatment or control groups (n = 5 per treatment group). Group allocation was performed by an investigator blinded to the treatment identity. Sham-treated mice served as controls for evaluating all experimental readouts.

Blinding

No blinding was applied to computational analyses, as these were performed using standardized and objective bioinformatics pipelines. In contrast, data collection and analysis for wet-lab and in vivo mouse experiments were conducted under blinded conditions. Most experiments were independently performed by two investigators, with at least one investigator blinded to group allocation.

Reporting for specific materials, systems and methods

We require information from authors about some types of materials, experimental systems and methods used in many studies. Here, indicate whether each material, system or method listed is relevant to your study. If you are not sure if a list item applies to your research, read the appropriate section before selecting a response.

Materials & experimental systems

n/a	Involved in the study	
☐	☒	Antibodies
☐	☒	Eukaryotic cell lines
☒	☐	Palaeontology and archaeology
☐	☒	Animals and other organisms
☒	☐	Clinical data
☒	☐	Dual use research of concern
☒	☐	Plants

Methods

n/a	Involved in the study	
☒	☐	ChIP-seq
☒	☐	Flow cytometry
☒	☐	MRI-based neuroimaging

Antibodies

Antibodies used

hnRNPL (4D11, Santa Cruz); GAPDH A19056,Abclonal); RBM3 14363-1-AP,Proteintech); NeuN 66836-1-Ig,Proteintech); Anti-DNA/RNA G-quadruplex [BG4] Ab00174-30.126, Absolute Antibody); Alexa Fluor® 488 anti-DYKDDDDK Tag Antibody 637317, BioLegend Also available in Supplementary data 2.

Validation

Validation data for each antibody are available on the manufacturers' websites, which can be found using the catalogue numbers provided.
The antibodies have also been validated in-house via knockdown and western blot experiments.

Eukaryotic cell lines

Policy information about cell lines and Sex and Gender in Research

Cell line source(s)

HEK293T (CRL-3216™, ATCC) and HeLa cells (CCL-2™, ATCC) , HEK293 cells (CL-0001; Procell Life Science & Technology Co., Ltd.), N2a CL-0168, Procell Life Science & Technology Co., Ltd.) and HT22 cells (iCell-m020; iCell Bioscience Inc.)

Authentication

All cell lines were authenticated by the suppliers at the time of purchase (Certificates of Analysis available) and further verified by short tandem repeat (STR) profiling. HEK93T and Hela are in the Heyd Lab for several years and are regularly checked for phenotype.

Mycoplasma contamination

All cell lines were routinely tested for mycoplasma contamination (every 3 months) and all tested negative.

Commonly misidentified lines
(See ICLAC register)

N/A

Animals and other research organisms

Policy information about studies involving animals; ARRIVE guidelines recommended for reporting animal research, and Sex and Gender in Research

Laboratory animals

Adult male C57BL/6J mice (20–25g, 8–10 weeks old) were obtained from the Animal Center of Wuhan University. All animals were on a C57BL/6J background.

Wild animals

N/A

Reporting on sex

Male

Field-collected samples

N/A

Ethics oversight

All mouse work was performed in accordance to the Institutional Animal Care and Use Committee (IACUC) of Wuhan University (IACUC issue No.: WDRM20240302B).

Note that full information on the approval of the study protocol must also be provided in the manuscript.

Plants

Seed stocks

Report on the source of all seed stocks or other plant material used. If applicable, state the seed stock centre and catalogue number. If plant specimens were collected from the field, describe the collection location, date and sampling procedures.

Novel plant genotypes

Describe the methods by which all novel plant genotypes were produced. This includes those generated by transgenic approaches, gene editing, chemical/radiation-based mutagenesis and hybridization. For transgenic lines, describe the transformation method, the number of independent lines analyzed and the generation upon which experiments were performed. For gene-edited lines, describe the editor used, the endogenous sequence targeted for editing, the targeting guide RNA sequence (if applicable) and how the editor was applied.

Authentication

Describe any authentication procedures for each seed stock used or novel genotype generated. Describe any experiments used to assess the effect of a mutation and, where applicable, how potential secondary effects (e.g. second site T-DNA insertions, mosiacism, off-target gene editing) were examined.
